# Phenotypic, Genomic, and Transcriptomic Comparison of Industrial Aspergillus oryzae Used in Chinese and Japanese Soy Sauce: Analysis of Key Proteolytic Enzymes Produced by Koji Molds

**DOI:** 10.1128/spectrum.00836-22

**Published:** 2023-02-06

**Authors:** Lijie Zhang, Le Kang, Yan Xu

**Affiliations:** a Laboratory of Brewing Microbiology and Applied Enzymology, Key Laboratory of Industrial Biotechnology of Ministry of Education, School of Biotechnology, Jiangnan University, Wuxi, Jiangsu, China; University of Nebraska—Lincoln

**Keywords:** *Aspergillus oryzae*, soy sauce, phenotypic analysis, comparative genome, comparative transcriptome, key proteolytic enzyme

## Abstract

Aspergillus oryzae, which generates numerous enzymes for the breakdown of raw materials, is an essential koji mold in soy sauce production. For better soy sauce productivity and flavor quality, China and Japan have developed their own industrial A. oryzae strains at distinct evolutionary branches for use in soy sauce production for decades. However, systematic comparison between the two national industrial strains has been poorly conducted, and thus we have not been able to generate adequate knowledge, especially regarding what are the key hydrolytic enzymes produced by A. oryzae during koji production. This study sequenced and assembled three high-quality genome sequences of industrial A. oryzae originating from China and Japan. Based on the genome sequences, a phylogenetic tree analysis was performed and revealed the evolutional distances between the two national industrial koji molds. Meanwhile, a comparative phenotypic analysis revealed that the two national industrial strains differed in growth and catalytic characteristics, particularly in proteolytic enzyme activities. To investigate the molecular mechanism underlying the phenotypic difference, we conducted systematic comparative genome and transcriptome investigations. We found minor differences in the quantity and diversity of proteolytic enzyme genes between Chinese and Japanese koji molds, while the protease secretion ratio and transcriptional level were dissimilar. We identified 58 potential important enzymes associated with high protein breakdown efficiency during industrial koji fermentation by combining comparative phenotypic and transcriptome data. More research is required to confirm the function of these putative key hydrolytic enzymes.

**IMPORTANCE** Aspergillus oryzae is widely used as an industrial koji mold for soy sauce brewing due to its powerful raw material decomposition capability. Although various proteases in A. oryzae have been identified, it remains a challenge to find essential enzymes involved in soy sauce production. Generally, the industrial A. oryzae used in soy sauce brewing has excellent proteolytic activity. Based on this, we analyzed key proteolytic enzymes according to a comparison of the genome and transcriptome between three industrial strains. This study found little difference in gene numbers and mutations of proteolytic enzymes between three industrial A. oryzae strains. However, variations in protease secretion ratio and transcriptome were discovered between industrial strains. Based on that, we generated 58 candidate key proteolytic enzymes. This work comprehensively analyzed three industrial koji molds, revealing genome development under separate artificial domestication and helping in the study of key proteolytic enzymes during soy sauce production.

## INTRODUCTION

Soy sauce, with its pleasant aroma and intense umami taste, is a widely used condiment in East Asia and is becoming popular in Western countries (1). Soy sauce is made in the following two stages: koji (solid-state) and moromi (submerged) fermentations (2, 3). The key microbe during koji fermentation is Aspergillus oryzae, which has powerful proteolytic and amylolytic activity for raw material breakdown (4, 5). Due to the importance of the A. oryzae strain, soy sauce manufacturers have been developing generations of industrial A. oryzae strains using diverse strategies such as UV or plasma mutagenesis (6–8). For steadily improving soy sauce productivity and flavor quality, more strategies, such as generating newer strains with more powerful decomposing capability, are urgently needed (9). However, knowledge of functional genes (particularly the key proteolytic enzymes) of A. oryzae is insufficient, making it difficult to build a more reasonable method for Koji's increased decomposing capability.

For investigating the characteristics of useful proteolytic enzymes, a dozen proteases, including neutral protease I (Npi), dipeptidyl peptidase, and Xaa-Pro aminopeptidase, etc., were homologously or heterologously expressed, purified, and analyzed (10–16). For example, Matsushita-Morita et al. investigated a novel Xaa-Pro aminopeptidase and described the characteristics of this enzyme, including molecular weight, optimum temperature, pH, and temperature stability (13). In 2016, Maeda et al., expressed, purified, identified, and compared three extracellular dipeptidyl peptidases (11). Although these enzyme properties can help us understand these *Aspergillus* protease families, they cannot answer the question of which key proteolytic enzymes are generated by A. oryzae during koji fermentation. In recent years, multi-omics strategies have been developed for the identification of key proteases (17–19). Zhong et al. analyzed the genomes and transcriptomes of an industrial strain of A. oryzae RD2 and strain TS2 with poor fermentative performance and identified putative genetic markers in A. oryzae associated with improved koji fermentation (17). Zhao et al. identified possible critical extracellular enzymes at several fermentation stages via extracellular proteome analysis (18). More research is needed to determine which enzymes play crucial roles during soy sauce brewing.

For centuries, the majority of soy sauce has been produced and consumed in China and Japan. Meanwhile, both countries have their own industrial koji molds. Industrial strains in the two countries have been domesticated independently for centuries. In China, the representative industrial koji mold of soy sauce is A. oryzae 3.042 (20). In Japan, although distinct koji molds are domesticated by different tane-koji manufacturers, these koji molds are categorized into the same branch in a high-quality phylogenetic tree, consistent with classifications by appearance and enzymatic activity (21). Interestingly, although different industrial strains show nonsynonymous and gap mutations in genes involved in fermentation characteristics after generations of domestication, they conserve industrially useful catalytic enzyme-encoding genes. Thus, the investigation of genes that are commonly involved in fermentation performance in industrial A. oryzae strains might help to characterize key enzymes involved in koji fermentation, especially when combined with phenotypic and transcriptional data.

In this study, we obtained high-quality genome sequences of one Chinese industrial strain A. oryzae 3.042 and two Japanese industrial A. oryzae strains, named LBM 30007 and LBM 30008, by using a combination of Illumina paired-end sequencing and Oxford Nanopore Technologies (ONT) sequencing. Then, combining publicly available genomic information, we assessed the evolutionary relationship of A. oryzae, which is used to make soy sauce (see Table S1 in the supplemental material). Furthermore, for the comparison study, genomic data and phenotypic data, such as growth parameters and fermentation performance, were combined, demonstrating that the number of hydrolase genes is not directly related to its degradation capacity. Finally, transcriptome sequencing (RNA-seq) analysis identified 58 candidate proteolytic enzyme genes that may play an important role during soy sauce production. This study systematically compared the phenotype, genome, and transcriptome of Chinese and Japanese soy sauce industrial A. oryzae, adding to our understanding of genome evolution through separate artificial domestication and aiding in the study of the key proteolytic enzymes during koji production.

## RESULTS

### Phenotypic comparison of industrial Aspergillus oryzae used in Chinese and Japanese soy sauce.

**FIG 1:**
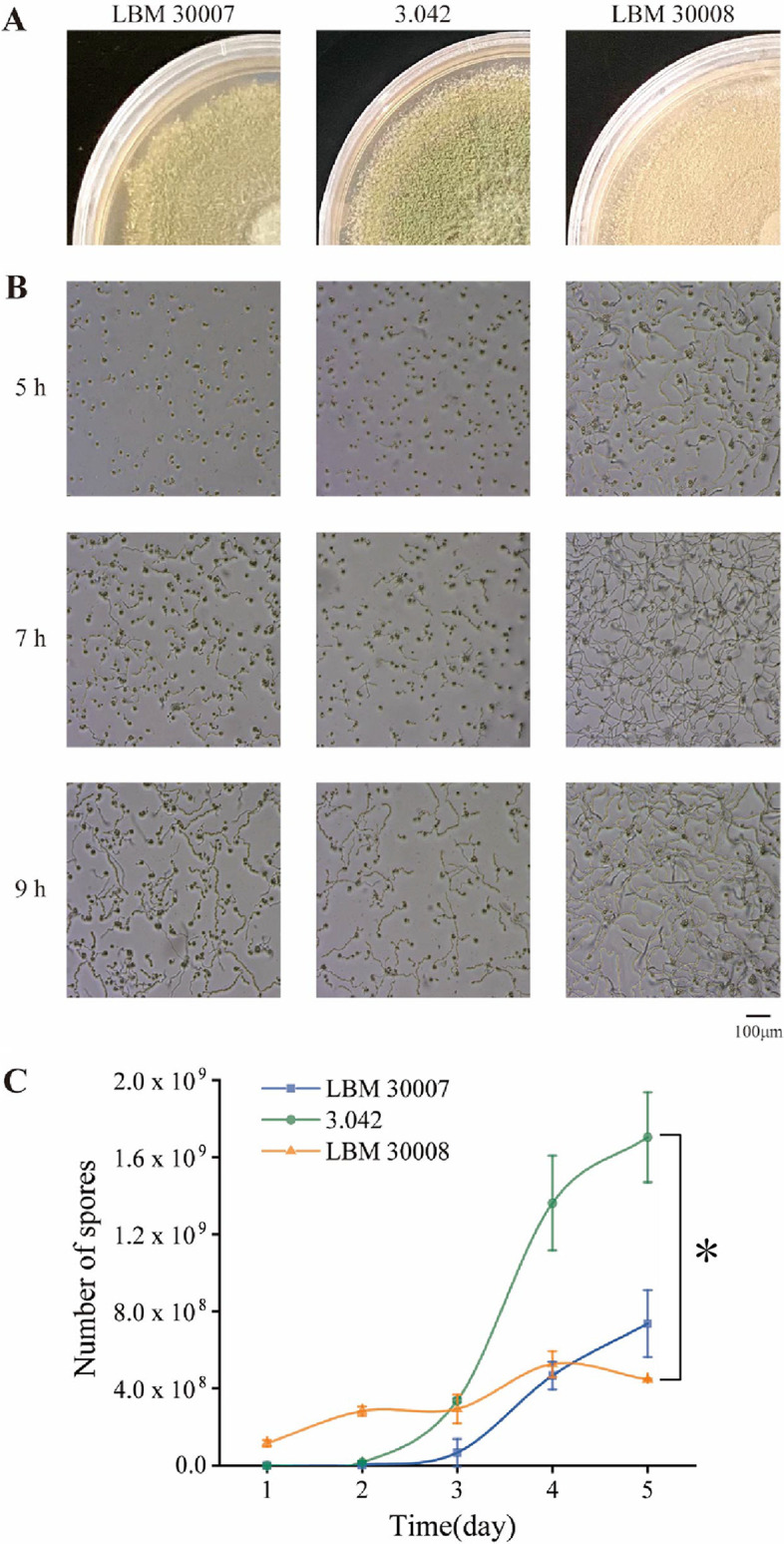
Growth traits of A. oryzae strains LBM 30007, 3.042, and LBM 30008 on solid medium. (A) Colony morphology. (B) Conidia germination and hyphae extension. (C) Number of spores produced by three A. oryzae strains. Data are presented as mean ± standard deviation. Significance analysis was determined by one-way ANOVA test and *post hoc*/multiple comparison test. *, *P* < 0.05.

In our lab collection, A. oryzae 3.042 is used for Chinese soy sauce production, and A. oryzae LBM 30007 and LBM 30008 are used for Japanese soy sauce production. In this work, we compared the phenotypes of Chinese and Japanese industrial A. oryzae, including morphological analysis, hydrolytic enzyme activity, spore production, and spore germination. As shown in Fig. 1A, the colony of 3.042 (cultivated on a potato dextrose agar [PDA] solid plate for 120 h) was Kronberg's green and floccose with neat and smooth white edges, whereas the colony of LBM 30007 (also cultivated on a PDA solid plate for 120 h) was light cress green, velvety, and had irregular edges. Meanwhile, LBM 30008’s colony was olive-yellow and velvety with neat edges.

Spore production and germination are the two primary fungal properties that received attention from koji manufacturers and soy sauce producers. During koji fermentation, koji mold with high spore production capability will increase seed koji productivity, while koji mold with rapid germination capability will lower the risk of bacterial contamination. As shown in Fig. 1B, LBM 30008 spores germinated in 5 h, making it the spore with the quickest germination capabilities, whereas strain 3.042 and LBM 30007 spores germinate in 7 h. Furthermore, after 5 days of incubation, strain 3.042 generates the most spores per gram (1.70 × 10^9^), whereas the Japanese koji mold LBM 30008 (4.46 × 10^8^) produces significantly fewer spores (Fig. 1C).

**FIG 2:**
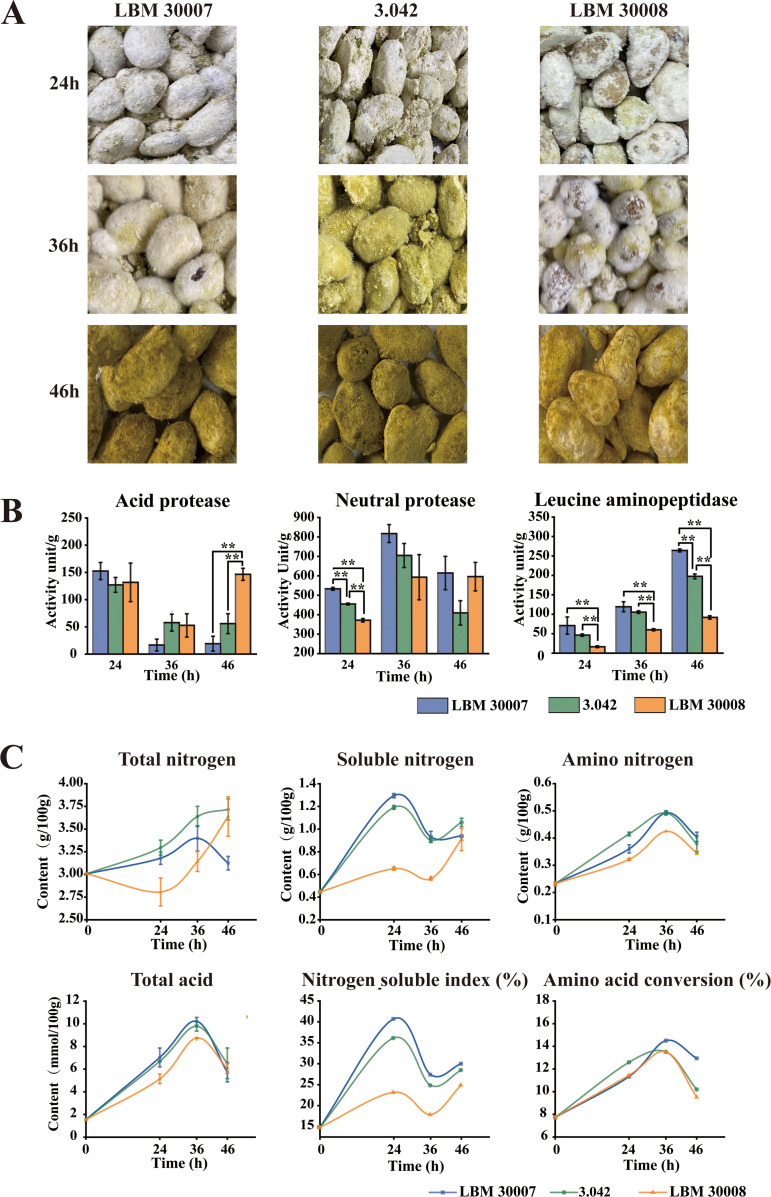
Phenotypes of soy sauce koji by using A. oryzae strain LBM 30007, 3.042, and LBM 30008 as the starter, respectively. (A) Growth phenotype. (B) Proteolytic enzyme activity. (C) Physicochemical property. Data are presented as mean ± standard deviation. Significance analysis was determined by one-way ANOVA test and *post hoc*/multiple comparison test. *, *P* < 0.05; **, *P* < 0.01.

To explore the fermentative performance of industrial A. oryzae more accurately, soy sauce koji was produced in the laboratory by using A. oryzae 3.042, LBM 30007, and LBM 30008 as starters (Fig. 2A). After 24 h of koji fermentation, all three koji molds formed white hyphae. After 36 h of koji fermentation, strain 3.042 generated visible yellow spores, whereas strains LBM 30007 and LBM 30008 also produced white hyphae. After 46 h of koji fermentation, koji matured with plump and yellow-green spores. The spore number of 3.042 in mature koji was significantly higher than that of LBM 30008. According to these findings, the Japanese koji molds A. oryzae LBM 30007 and 30008 have a longer mycelium development time, while the Chinese koji mold A. oryzae 3.042 has a longer spore formation period and a more powerful spore formation capability.

The most important characteristic of industrial koji molds is their protease activities. According to Fig. 2B, neutral protease showed the maximum enzymatic activity at 500 to 700 U/g, followed by acidic protease at 50 to 150 U/g and leucine aminopeptidase at 50 to 250 U/g. During the 46-h koji fermentation process, strain LBM 30007 has the highest neutral protease activity, whereas strain LBM 30008 has the lowest. The activity of leucine aminopeptidase, a type of zinc-requiring metalloprotease, was also measured. Leucine aminopeptidase has been shown to effectively reduce the bitterness of soy sauce through the removal of the hydrophobic amino acid residues from the N terminus of peptides (22). As shown in Fig. 2B, leucine aminopeptidase activity increased along with the fermentation time. Strain LBM 30007 has significantly higher leucine aminopeptidase activity than LBM 30008 and 3.042.

In addition to proteolytic enzymes, we also measured other enzyme activities. Pectinase dissolves the cell wall and aids in the release of protein and starch from the raw material (6). As shown in Fig. S1 in the supplemental material, pectinase activity was high in the initial phase and then decreased in the following koji fermentation stages. All three industrial strains have similar pectinase activity at 24- and 46-h fermentation times. Amylase and glucoamylase are two key enzymes for starch hydrolysis. Both enzyme activities increased during the koji fermentation time. In addition, LBM 30007 has the highest enzyme activity for starch hydrolysis. Compared with the koji sample made by 3.042, koji samples made by Japanese strains had higher carbohydrase activity and higher glucose content (see Fig. S2 in the supplemental material).

In this study, we produced soy sauce koji on a laboratory scale using various industrial strains as koji molds. In comparison to LBM 30007, the koji sample inoculated with strain 3.042 had higher concentrations of soluble nitrogen after 46 h of fermentation (Fig. 2C; see also Fig. S3 in the supplemental material). At 24 h, strain LBM 30007 had the highest nitrogen soluble index of 40.73%, while those of strains 3.042 and LBM 30008 were 36.15% and 23.18%, respectively. Amino nitrogen is an important indicator of soy sauce. As shown in Fig. 2C, the concentration of amino nitrogen in koji produced with LBM 30007 as the starter was slightly higher than that of strain 3.042. Compared to the other two industrial strains, LBM 30008 produced koji with a lower concentration of amino nitrogen (Fig. S3).

In conclusion, A. oryzae 3.042, which is used in Chinese soy sauce production, grows faster and has enhanced sporulation capacity. Strain LBM 30007 shows more hydrolase enzyme activity and a comparable nitrogen solubility index to strain 3.042. Strain LBM 30008 needs the least time for the spore to grow, but it doesn't break down proteins very well. From the perspective of fermentative performance, these three industrial koji molds differ significantly. Thus, comparative genomes that can explain the molecular mechanism underlying these performance differences are urgently required.

### Genome characteristics of industrial A. oryzae strains.

In this study, we sequenced and assembled three industrial strains, namely, A. oryzae 3.042, A. oryzae LBM 30007, and A. oryzae LBM 30008, by combining Illumina short reads (Table S1 and Table S2 in the supplemental material) and Oxford Nanopore Technologies (ONT) long reads (see Table S3 in the supplemental material). After quality trimming, adapter removal, and contaminant filtering, we obtained clean data, which was then used to assemble high-quality genomes (Table 1; see also Tables S4 to S7 in the supplemental material). According to Table 1, A. oryzae 3.042 has a minimum genome size of 37.18 Mb and 15 scaffolds. The genomic lengths of LBM 30007 and LBM 30008 are 38.1 Mb and 39.05 Mb, respectively, and their scaffold numbers are 18 and 25, respectively. These genome sizes are comparable to those previously reported (23). Results of the BUSCO alignment showed that the proportion of fully assembled genes in the final assembly genomes of the three A. oryzae strains is 99.3%, while the proportion of missing sections is 0.7% for LBM 30007 and 3.042 and 0.4% for LBM 30008. In addition to BUSCO analysis, we also used the complete set of 248 core eukaryotic genes (CEGs) to do CEGMA prediction for assessing the quality of the genome assembly. For LBM 30007, 237 of the 248 (95.56%) genes entirely matched our assembled genome, which is somewhat fewer than the 238 (95.97%) genes of 3.042 and LBM 30008. Moreover, we analyzed the genome synteny blocks and discovered that, with the exception of minor rearrangements, the three strains sequenced in this study have more sequence coverage than the model strain A. oryzae RIB40 (see Fig. S4 in the supplemental material). All of the results of BUSCO alignment, CEG analysis, and synteny blocks indicated that the genome assembly in this study is high quality, complete, and accurate.

**TABLE 1:** Summary of genome assembly and annotation of three soy sauce industrial A. oryzae strains LBM 30007, 3.042, and LBM 30008

Feature	A. oryzae strain		
	LBM 30007	3.042	LBM 30008
Assembly feature			
Scaffolds no.	18	15	25
Total bases in scaffold (bp)	38,099,469	37,176,367	39,048,948
Largest scaffold length (bp)	6,370,115	6,621,566	6,331,409
Scaffold *N*_50_ (bp)	2,759,338	2,723,563	2,398,028
BUSCOs (%)			
Complete	99.3	99.3	99.3
Complete duplicated	0.7	0.7	0.7
Fragmented	0.0	0.0	0.3
Missing	0.7	0.7	0.4
CEGs (%)			
Assembled	97.18	97.58	97.58
Completely assembled	95.59	95.97	95.97
Genome annotation (Maker2)			
Predicted coding gene no.	13,191	12,615	13,131
Predicted proteins avg length (bp)	1,720.66	1,787.14	1,814.38
Gene density (kb)	0.35	0.34	0.34
Gene/genome (%)	59.57	60.64	61.01
Intergenetic length/genome (%)	40.43	39.36	38.99
Functional annotation			
NR (%)	95.48	96.10	95.91
KOG (%)	77.96	79.34	78.24
Pfam (%)	71.85	73.28	71.99
GO (%)	69.50	70.69	69.29
Swiss-Prot (%)	65.51	67.42	65.90
KEGG (%)	29.20	29.77	28.73

We obtained potential protein-coding genes by using the annotation pipeline MAKER2 (Table 1; see also Table S7). Consistent with the genome size, A. oryzae 3.042 has the fewest number of genes, with 12,615, compared to those of LBM 30007 and LBM 30008, which have 13,191 and 13,131 genes, respectively. It is worth noting that LBM 30007 has the most genes and has the highest gene density of 0.35 compared to that of 3.042 and LBM 30008, both of which have a gene density of 0.34. LBM 30007 has a gene length of 22.70 Mb, whereas the average gene length is 1,721 bp. The average gene length for strain 3.042 is 1,787 bp, with a total gene length of 22.54 Mb. Strain LBM 30008 has the longest average gene length of 1,814 bp and the longest gene length of 23.82 Mb. Moreover, as shown in Table 1 and Tables S8 to S10 in the supplemental material, we performed gene functional annotation using diverse databases, such as eggNOG, GO, Pfam, Swiss-Prot, KEGG, and NCBI, to guarantee that the vast majority of genes could be annotated by at least one database.

### Phylogenetic relationship.

**FIG 3:**
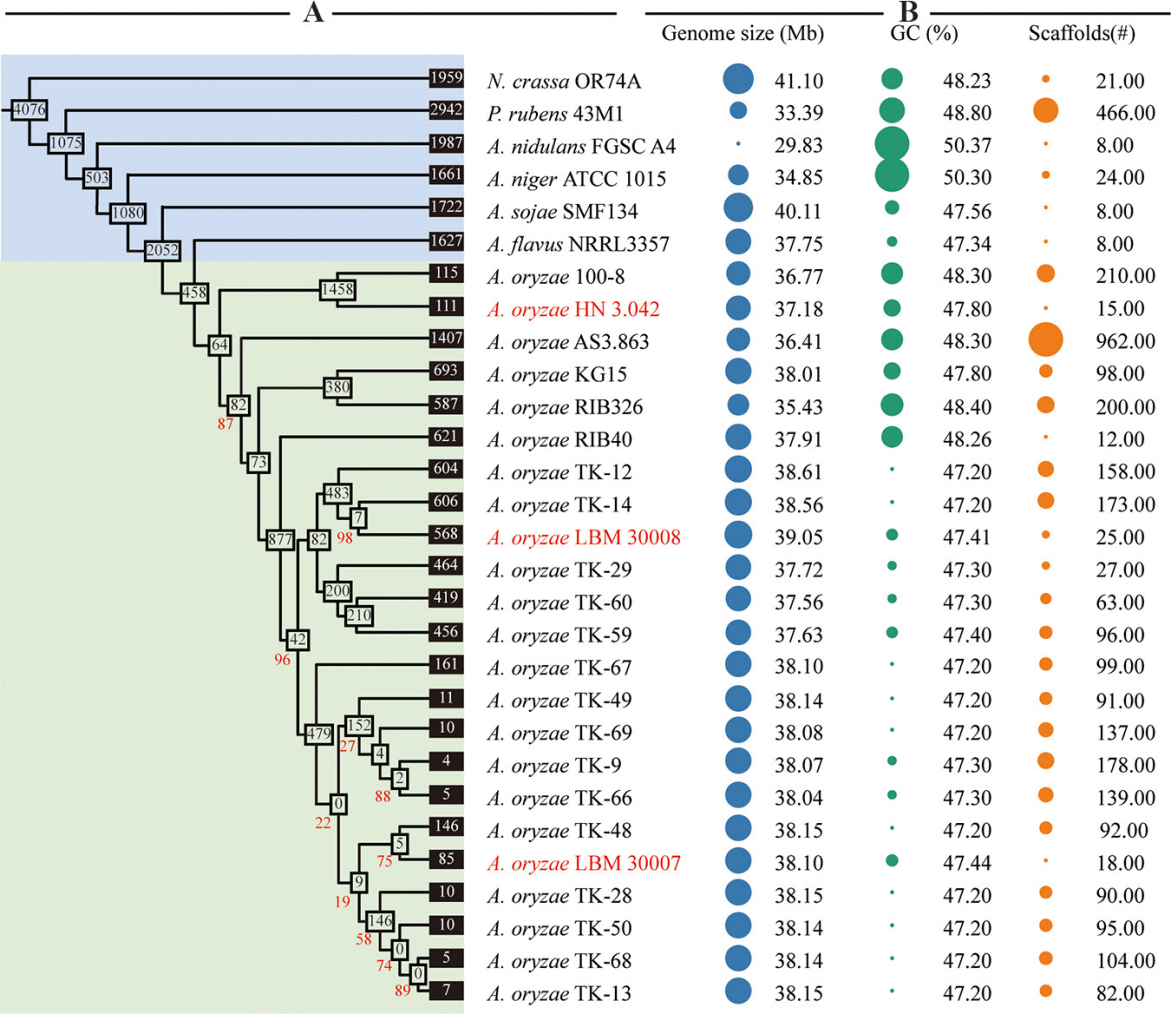
Phylogeny and genome statistics of industrial A. oryzae that are used for soy sauce production. (A) A maximum likelihood phylogenomic tree was constructed using OrthoFinder, MAFFT, Gblocks, SeqKit, and IQ-TREE based on entire concatenated single-copy orthogroups. For more clarity and intuitiveness, we ignored branch lengths. The red numbers at the nodes indicate the bootstrap support of the particular branch. Unlabeled nodes have 100% bootstrap support. The white boxes in the nodes represent the orthogroups shared among the species branch. The black boxes at the tips represent the orthogroups unique to that individual species. The fuchsia letters indicate the A. oryzae strains sequenced in this study. (B) The bubble chart shows the genome characteristics and sequencing parameters. The size of the bubble has been scaled for each panel and is not comparable across panels.

To investigate the evolutionary relationships between A. oryzae strains used for soy sauce production, including the three A. oryzae strains analyzed in this study, a total of 22 genomes of soy sauce industrial A. oryzae were obtained (see Table S11 in the supplemental material). In addition, seven reference genomes were collected, of which A. oryzae RIB40 was the model strain of A. oryzae (Fig. 3; genome link shown in Table S11). Among them, 16 A. oryzae strains are used for the fermentation of soy sauce in Japan, whereas two strains and one strain are employed for the production of soy sauce in China and Korea, respectively. Then, a comparative genome analysis of 29 fungal strains was first conducted using OrthoFinder software. We obtained 12,757 orthogroups from 327,659 proteins, while 3,467 proteins were unclustered. Then, to investigate the evolutionary relationship and divergence of industrial A. oryzae strains, a phylogenetic tree was constructed by using concatenated single-copy orthologous proteins.

As shown in Fig. 3A, the collected A. oryzae strains formed one branch of the phylogenetic tree. Furthermore, the phylogenetic tree distinguishes between industrial A. oryzae strains from different countries. Chinese industrial strains, including A. oryzae 3.042, 100-8, and AS 3.863, were clustered into one group. Similarly, 19 industrial Japanese strains were grouped in a separate cluster. This finding confirmed that the strains LBM 30007 and LBM 30008 previously stored in our strain collection were Japanese koji molds. In addition, when the data from phenotypic comparison and phylogenetic analysis were combined, we discovered numerous genetic and phenotypic differences between Chinese and Japanese industrial strains, even though all of these strains possess excellent characteristics for soy sauce production in their respective countries. Thus, investigating the individual and shared genes of three industrial strains might help us to identify the candidate key enzymes associated with the high productivity of soy sauce.

Aspergillus flavus has long been recognized as the “evil twin” of A. oryzae (24). According to the phylogenetic tree, A. oryzae is most closely linked to A. flavus, and they share 9,244 orthogroups, supporting that A. flavus and A. oryzae are genetically close species. Researchers have focused their attention on the possibility of aflatoxin generation by A. oryzae since A. flavus may create highly carcinogenic aflatoxin (25). This work studied and analyzed the aflatoxin production gene clusters in industrial strains of A. oryzae. As shown in Fig. S5 in the supplemental material, the aflatoxin-producing strain A. flavus NRRL 3357 has the complete aflatoxin-forming gene cluster. Different from NRRL 3357, the aflatoxin biosynthesis gene clusters in industrial A. oryzae can be divided into four categories, with the 3.042 and TK-29 types being the most prevalent. Because of the incompletion of the gene cluster, TK-29-type A. oryzae is unlikely to produce aflatoxin. In addition, although 3.042-type A. oryzae has considerably more complete aflatoxin biosynthesis gene clusters than the TK-29 type, its gene *aflJ* has amino-acid substitutions compared to A. flavus (aflatoxigenic), which inhibits the function of AflJ. The inactivation of AflJ will prevent the protein AflR from performing its function (26). Thus, 3.042-type A. oryzae was incapable of synthesizing aflatoxin, which is consistent with the detection data obtained through ultraperformance liquid chromatography-electrospray ionization triple quadrupole mass spectrometry (UPLC-QQQ-MS) (see Fig. S6 in the supplemental material). In summary, all of the detected industrial A. oryzae strains related to soy sauce production have no risk of aflatoxin biosynthesis, consistent with previous reports. In addition, from the perspective of aflatoxin biosynthesis gene clusters, the Chinese industrial strain was likewise grouped in a distinct clade from the Japanese industrial strain, demonstrating the accuracy of the phylogenetic tree generated in this study.

### SNP and InDel analysis.

**FIG 4:**
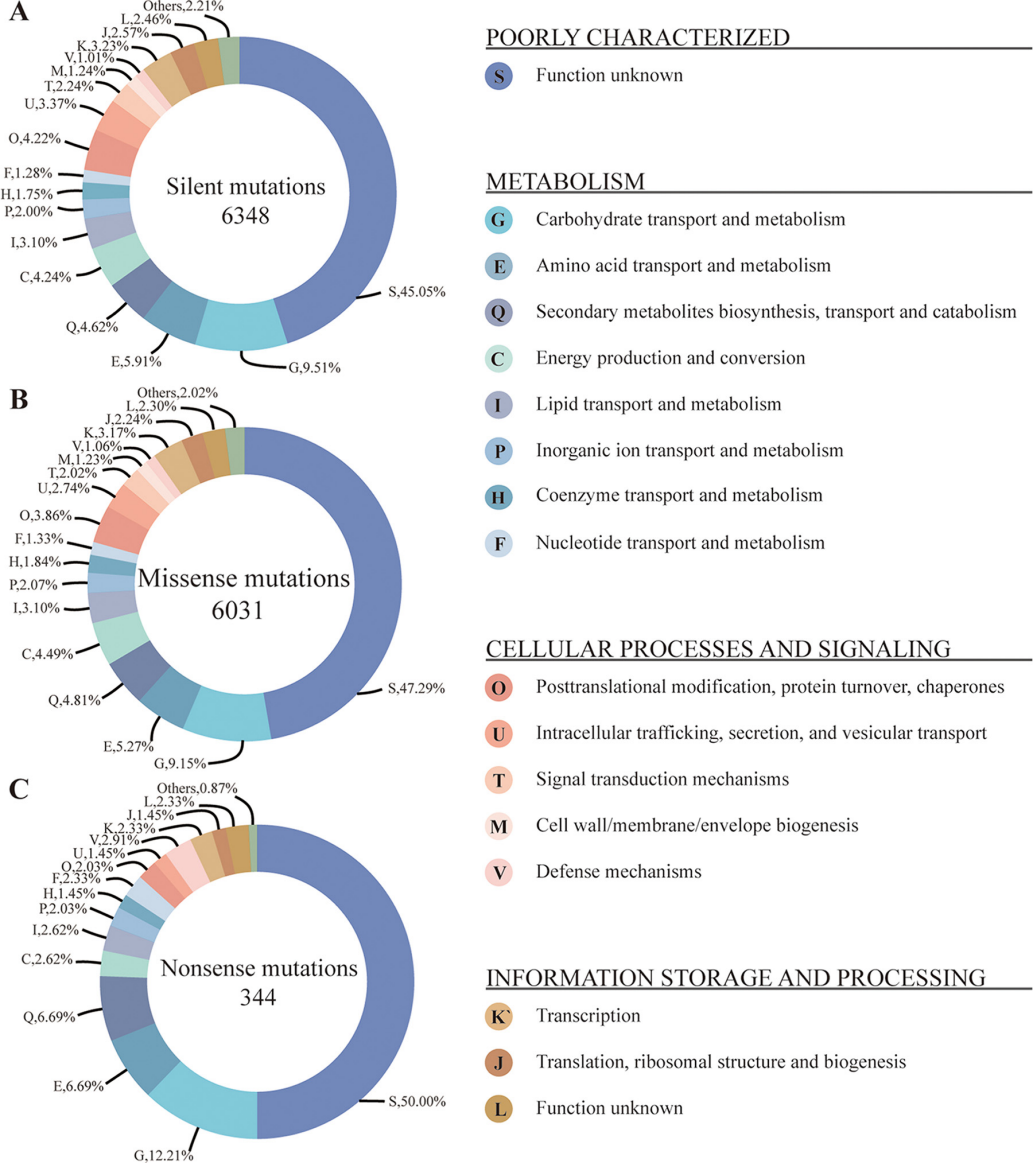
Functional distribution of the SNP variant genes between Japanese industrial A. oryzae LBM 30007/LBM 30008 and Chinese industrial A. oryzae 3.042. (A) Silent mutations. (B) Missense mutations. (C) Nonsense mutations. The numbers in the center of these circles represent the total number of annotated gene variants. The percentage of eukaryotic orthologous groups (KOG) categories, which is less than 1%, was not shown.

As shown above, although these three strains are all industrial strains used for soy sauce production, strains 3.042, LBM 30007, and LBM 30008 belong to distinct clades in the phylogenetic tree, consistent with their unique fermentation characteristics (especially diverse protease activities). We wanted to investigate how many and what genes are mutated between these three industrial strains and whether and how many of the mutated genes are associated with protein decomposition. In this study, we analyzed single nucleotide polymorphism (SNP) and short genomic insertion and deletion (InDel) between Japanese strains A. oryzae LBM 30007 and LBM 30008 and Chinese strain A. oryzae 3.042, with A. oryzae 3.042 serving as the control strain. After filtering the raw data, we generated 177,564 SNPs and 22,984 InDels, respectively. Approximately 2.04% of the InDels are assumed to have a significant (disruptive) effect on the protein, whereas 96.95% are usually noncoding variations or variants affecting noncoding DNA. In addition, as shown in Fig. 4, 52.96% of the SNPs (6,348 genes) are synonymous, and 46.23% of them are missense (6,031 genes), while the remaining 0.81% are nonsense (344 genes).

We annotated the SNP variant genes by using the eggNOG database. The annotation rates for silent mutations and missense mutations are 54.95% (3,488 genes) and 52.71% (3,179 genes), respectively (Fig. 4A and B), while the annotation rate for nonsense mutations is 50.00% (172 genes) (Fig. 4C). Furthermore, the annotated genes were categorized according to their different gene functions. As shown in Fig. 4, most SNP mutated genes are primarily associated with categories G (carbohydrate transport and metabolism), E (amino acid transportation and metabolism), and Q (secondary metabolites biosynthesis, transport, and catabolism), which are closely associated with raw material utilization and flavor compound metabolism. In addition, the SNP variant genes were annotated by the GO database, and the results of the GO enrichment study are consistent with those of the eggNOG database (see Fig. S7 in the supplemental material). As shown in Table S12 and S13 in the supplemental material, two nonsense mutation genes and 25 missense mutation genes are proteolytic enzymes. These results showed that more than 6,000 genetic changes existed between Chinese and Japanese industrial A. oryzae strains and that 27 genes have a role in proteolysis, which might be closely connected to the productivity of soy sauce koji production.

### Strain-specific genes.

**FIG 5:**
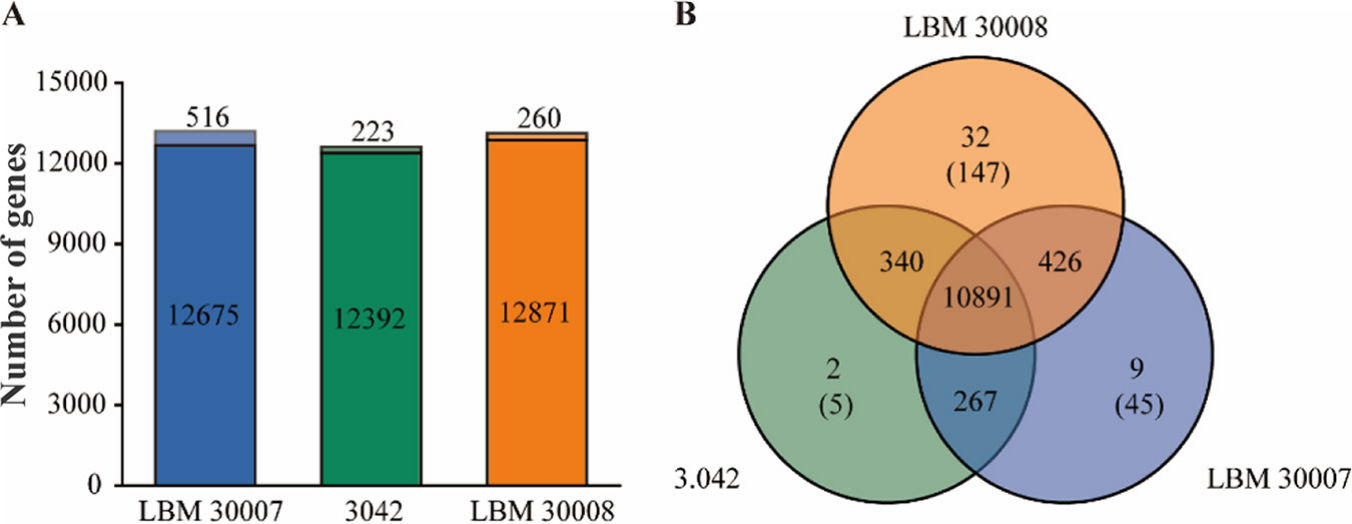
Comparative genome analysis of A. oryzae LBM 30007, 3.042 and LBM 30008. (A) The barplot shows the number of clustered genes (dark color) and unclustered genes (light color) during comparative genome analysis. (B) The Venn diagram represents the number of shared/specific orthogroups, and the number in parentheses represents the number of strain-specific genes.

Comparative genomic analyses were performed in this study to investigate in-depth genetic variations between the sequenced industrial strains. As shown in Fig. 5A, most genes are effectively clustered, suggesting that the subsequent strain-specific gene analyses are credible. After the OrthoFinder analysis, 11,967 orthogroups were identified and constructed, of which 91.02% (10,891 orthogroups) were present in all three A. oryzae strains (Fig. 5). Furthermore, LBM 30008 possesses the most strain-specific genes (147 genes). In contrast, strains LBM 30007 and 3.042 contain 45 and 5 strain-specific genes, respectively (Fig. 5B). To determine if strain-specific genes are associated with soy sauce fermentation performance, we analyzed the genetic functions of these unique genes.

As shown in Table S14 in the supplemental material, A. oryzae 3.042 possesses three prolyl 4-hydroxylase-related genes (3.042_gene03909, 3.042_gene03910, and 3.042_gene03911), each of which catalyzes the posttranslational formation of 4-hydroxyproline and causes a reduction in the content of the sweet amino acid proline. In addition, the 3.042 genome contains two retrotransposon-derived PEG10 proteins (3.042_gene02262 and 3.042_gene08653) that have been widely researched in mammals and play a role in cell proliferation, differentiation, and apoptosis resistance (27). Both strains LBM 30007 and LBM 30008 possess a significant number of transposase genes, which may induce chromosomal breakage at a specific region and are responsible for genomic DNA rearrangements (28). Transposase has been linked to multiple copies of α-amylase in A. oryzae, and it is predicted to contribute to beneficial genetic changes (29). LBM 30008 specifically has six drug resistance genes (KL-4_gene11039, KL-4_gene11740, KL-4_gene12930, KL-4_gene13195, KL-4_gene13338, and KL-4_gene13348). These strain-specific genes can be directly or indirectly related to the fermentation performance of A. oryzae, thereby explaining its adaptability to the environment or high strain specificity. However, these strain-specific genes seldom accounted for the differential proteolytic activity of three industrial koji molds.

### Proteolytic enzymes.

Different proteolytic enzyme genes in A. oryzae have complementary roles. Endopeptidases (alkaline protease and neutral proteases I and II) and exopeptidases (leucine aminopeptidases I and II) contribute to the solubilization of raw proteins during soy sauce production, whereas leucine aminopeptidases and acid carboxypeptidases are associated with the release of umami-amino acid-glutamic acid (18, 30). As shown above, the protease profiles and activities of three industrial strains are very different, but SNP, InDel, and strain-specific gene analyses found very few differences in protease genes.

Then, we used several databases to search for the profile and number of proteolytic enzyme genes based on their functional annotation. As shown in Table 2, there are 149 proteolytic enzyme genes in A. oryzae 3.042, while there are 154 and 155 in A. oryzae LBM 30007 and LBM 30008. We assumed that the number of protease genes might not be the most important thing that affects enzyme activity since strain LBM 30008 has the least proteolytic enzyme activity and the most protease genes. In fact, extracellular protease can help break down raw materials (macromolecules) more than intracellular protease. In addition, the signal peptide is the most common way to support enzyme secretion. Therefore, we determined whether and how many signal-peptide-containing proteases are in different industrial strains by using SignalP software. As shown in Table 2, strain LBM 30007 has the most proteolytic enzymes that contain signal peptides (63), which could be one reason why LBM 30007 has the highest proteolytic enzyme activity during the koji fermentation stage (Fig. 2B).

**TABLE 2:** Numbers of proteolytic enzyme genes in A. oryzae strains LBM 30007, 3.042, and LBM 30008^*a*^

Enzyme gene	EC no.	LBM 30007	3.042	LBM 30008
Exopeptidase				
Aminopeptidase	3.4.11.-	24/4	23/2	24/2
Dipeptidase	3.4.13.-	2/0	3/0	4/0
Dipeptidyl or tripeptidyl peptidase	3.4.14.-	7/3	11/4	11/4
Serine-type carboxypeptidase	3.4.16.-	14/11	13/8	14/10
Metallocarboxypeptidase	3.4.17.-	14/7	13/6	11/6
Omega peptidase	3.4.19.-	2/0	1/0	2/0
Total		63/25	64/20	66/22
Endopeptidase				
Serine protease	3.4.21.-	25/11	25/7	26/6
Cysteine proteinase	3.4.22.-	14/0	11/0	12/0
Aspartic endopeptidase	3.4.23.-	15/14	16/13	15/12
Metallopeptidase	3.4.24.-	28/9	27/4	28/5
Total		82/34	79/24	81/23
Unknown		9/4	6/3	8/4
Exopeptidases + endopeptidases		154/63	149/47	155/49

a The number before the slash indicates the number of proteolytic enzyme genes in the genome, and the number after the slash represents the number of genes with the signal peptide predicted by SignalP.

In this study, RNA-seq was used to investigate the transcriptional level of proteolytic enzyme genes in three industrial A. oryzae strains at different fermentation stages in order to describe additional features impacting proteolytic capacity and to analyze key proteolytic enzymes during koji fermentation. A. oryzae RIB40 is the model strain of this species, as it possesses genomic information at the chromosome level and thus serves as the model strain for transcriptional information analysis.

After transcriptional data alignment, more than 90% of transcriptional reads could be mapped to the type strain A. oryzae RIB40 genome. In 2007, Japanese researchers predicted that the A. oryzae RIB40 genome has 134 peptidase genes, which account for around 1% of the total genes in its genome (31). In this study, using the RIB40 genome as a model library, we annotated the transcriptomes of three commercial strains of A. oryzae and found a total of 155 genes encoding proteolytic enzymes (see Fig. S8 in the supplemental material).

Based on transcriptional levels, 97 genes were identified as differentially expressed genes (DEGs). In addition, a number of genes were selected at random to serve as examples, and the reverse transcription-quantitative PCR (qRT-PCR) method was used to confirm the transcript levels of these genes (see Fig. S9 in the supplemental material). If we regarded the expression difference of the same A. oryzae strain at different koji fermentation stages as a “horizontal difference” and the expression difference of different A. oryzae strains at the same fermentation stage as a “vertical difference,” we discovered 50 horizontal differences and 89 vertical differences between three industrial A. oryzae strains at varied fermentation periods. There are 58 proteolytic enzyme genes whose expression is similar. In addition, genes with low transcriptional levels are thought to have little to do with soy sauce production. We believe that the proteolytic enzymes that play an important role in the soy sauce brewing process consist of two distinct components as follows: (i) genes with high transcript abundance in non-DEGs, and (ii) genes with relatively high transcript abundance in both A. oryzae LBM 30007 and 3.042 at the same time in vertical DEGs.

**FIG 6:**
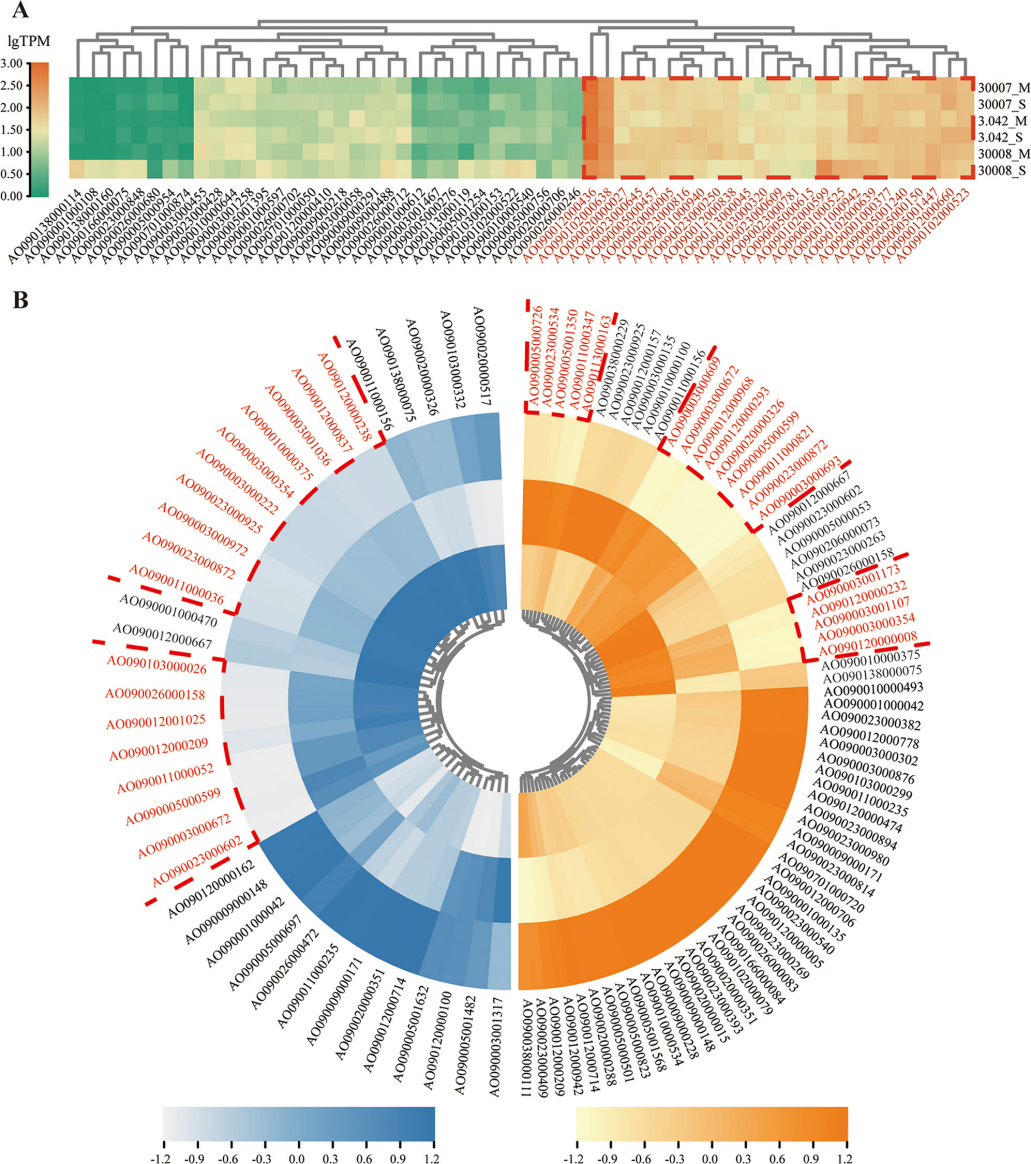
Heatmaps from hierarchical clustering of three A. oryzae genes, which encode proteolytic enzymes at different koji fermentation stages. Genes marked in red are predicted proteolytic enzyme genes that may be critical for soy sauce production. (A) Nondifferentially expressed genes. M, mycelium expansion stage; S, sporulation stage. (B) Vertical differentially expressed genes. The circles from inside to outside represent the transcription of A. oryzae LBM 30007, 3.042, and LBM 30008, respectively. Normalize the transcription abundance of each gene. (Left) Mycelium expansion stage; (Right) sporulation stage.

Therefore, we proposed 58 proteolytic enzymes that may be essential for soy sauce brewing, including 25 non-DEGs and 33 vertical DEGs (Fig. 6; see also Table S15 in the supplemental material). Many genes encoding proteolytic enzymes have been considered essential by an earlier study. For example, *alp1*/AO090003001036 is closely related to the solubility of soy sauce moromi, and *lapA*/AO090011000052 and lap2/AO090003000354 effectively reduce bitterness by removing the hydrophobic amino acid residues at the N terminus of the polypeptide (5, 22). Concurrently, we uncovered a large number of previously unrecognized hydrolase genes. For example, AO090023000872, which is annotated as pepsin-like aspartic protease, works under acidic conditions, and AO090005001240 is predicted to have the activity of Xaa-Pro aminopeptidase. These results give suggestions for future studies on which proteolytic enzymes play a significant role in soy sauce production.

## DISCUSSION

Aspergillus oryzae was first isolated from koji by H. Ahlburg in 1876 (4). Because of its high hydrolase activity and strain safety, it quickly replaced other starters as the primary starter in traditional fermented foods. In addition to being a saccharifying and diastatic agent, koji also adds to the color, taste, and aroma of fermented foods. The quality of koji greatly depends on the properties of koji molds. Thus, it is recognized that fermented food makers have an urgent need for high-quality koji molds. A. oryzae, which is typically utilized as koji mold, has been recognized as the national microorganism of Japan, similar to the status of the sakura (cherry blossom) as the national flower (32).

As the national microorganism, generations of industrial A. oryzae have been domesticated in Japan. Although the phylogenetic tree demonstrated that Japanese industrial A. oryzae clustered into one evolutionary branch (Fig. 3), it is challenging to identify a representative strain for Japanese industrial koji molds (21). As shown in Fig. 3, A. oryzae LBM 30007 and LBM 30008 grouped in a distinct clade, which was consistent with their fermentative performance. As an industrial strain, A. oryzae LBM 30008 has the fastest spore germination rate and produces spores at the earliest, but its hydrolase activities are relatively low. On the contrary, A. oryzae LBM 30007 has a superior advantage in hydrolase activity, while its spore germination rate is significantly slower than LBM 30008. Even though A. oryzae LBM 30007 and 30008 are both Japanese industrial strains, their phenotypes and genotypes differ greatly. Consequently, both strains may be the representative strains for Japanese soy sauce koji molds.

Chinese soy sauce industrial A. oryzae strains, including A. oryzae AS 3.863, A. oryzae 100-8, and A. oryzae 3.042, were clustered into one branch (Fig. 3). This result is inconsistent with a previous report (21). In earlier studies, A. oryzae AS 3.863, previously used for soy sauce production in China, was located on different evolutionary branches from other soy sauce industrial koji molds. A. oryzae 3.042, with increased hydrolase activity, is the UV-mutated daughter cell of strain AS 3.863 (33). A. oryzae 100-8, with high acid protease capacity, was generated from strain 3.042 by N^+^ ion implantation (34). Thus, the three Chinese koji molds should be located on the same evolutionary branch theoretically. Despite claims that A. oryzae 100-8 has more acid protease capabilities, A. oryzae 3.042 is basically the only industrial koji mold used in Chinese soy sauce. Combining the close evolutionary relationship of three Chinese industrial strains, we selected A. oryzae 3.042 as the only representative strain for Chinese soy sauce koji molds.

The ideal phenotype is an important reference factor for the domestication and selection of industrial strains. Rapid growth rate, high mycelial content, great sporulation capacity, and strong hydrolytic enzyme activities (particularly high proteolytic enzyme activities), are desired characteristics of A. oryzae for soy sauce brewing (5). In this study, we first evaluated the growth characteristics and fermentation performance of three industrial A. oryzae strains utilized in the production of Chinese and Japanese soy sauce. Based on germination time and mycelial elongation rate, we determined that the duration of the vegetative growth phases of LBM 30007 and 3.042 are homologous. Different from vegetative growth, strain 3.042 sporulates earlier and generates more spores than LBM 30007 and LBM 30008 (Fig. 1). Furthermore, strain LBM 30008 has the fastest spore germination rate and germinate spores at the earliest. LBM 30008's fast spore germination ability may protect industrial koji from bacterial contamination. In other words, LBM 30007's strong hydrolase activities may aid in the effective breakdown of raw materials during koji fermentation. This study suggested that industrial strains may not necessarily possess all “ideal” characteristics. Thus, instead of trying to create a “perfect” koji mold strain, we can learn a lot from the research into the fermentative performance of industrial strains and use that information to develop new methods for higher soy sauce productivity and flavor quality.

The biosynthetic risk of mycotoxin in A. oryzae, particularly aflatoxin, has been a research focus (35, 36). In this study, we analyzed the aflatoxin biosynthesis gene clusters in A. oryzae for soy sauce production and discovered that these A. oryzae have no risk of aflatoxin synthesis based on gene cluster analysis and fermentative validation (see Fig. S6 in the supplemental material). In addition, we found that the gene clusters of aflatoxin production in A. oryzae may be separated into four categories (see Fig. S4 in the supplemental material) as follows: RIB326 type, TK-29 type, 3.042 type, and TK-60 type (25).

In previous studies, molecular biological operations such as gene knockdown, gene knockout, and knock-in, as well as homologous or heterologous expression of proteolytic enzymes, were used to detect the proteolytic enzyme functions in A. oryzae (6, 10–16). However, it is still unclear which are the key proteolytic enzymes during soy sauce production, which hinders the rational design of next-generation strategies for higher soy sauce productivity. This study compared the phenotype and genetic relationship of A. oryzae used in Chinese and Japanese soy sauce production. We found obvious differences in the proteolytic enzyme activities of three industrial strains, and we wish to determine the genetic differences underlying this phenotype difference, particularly the significant difference in proteolytic enzyme activity between three industrial strains (A. oryzae 3.042 used in China and LBM 30007 and LBM 30008 in Japan). According to SNP and InDel analyses, there are few missense mutations in genes affecting carbohydrate and amino acid metabolism. In addition, the number of genes relevant to protein degradation in the three industrial strains is identical. This study revealed that gene numbers do not necessarily correspond to the brewing phenotype, which is consistent with earlier research (37).

LBM 30007 and LBM 30008 contain a large number of transposase genes that may influence gene expression. Thus, comparative transcriptome analysis was then conducted in this study. By comparing the transcription of proteolytic enzymes in A. oryzae at different koji fermentation stages, we discovered 58 candidate key enzymes. Numerous protease genes, including alkaline protease gene *alp1*/AO090003001036, neutral protease gene *NpI*/AO090011000036, leucine aminopeptidase gene *lapA*/AO090011000052, *lap2*/AO090003000354, dipeptidyl peptidase gene *dppB*/AO090023000602, serine-type carboxypeptidases gene *CpI*/AO090103000026, and aspartyl aminopeptidase gene *dapA*/AO090005001447, played important roles in soy sauce brewing, such as degrading soybean protein, removing bitterness, and increasing freshness, etc. (5, 38).

Furthermore, we discovered many key proteolytic enzyme genes that have not been investigated before and may possibly play important roles in soy sauce production. For example, this study discovered AO090023000872, annotated as a pepsin-like acid protease, which may play a crucial role in koji fermentation. As we all know, pH will decrease along with koji fermentation. When the koji fermentation reaches the middle or late stages, the pH decreases to around 4.5, inhibiting the activity of neutral and alkaline proteases. Consequently, acid proteases may be important for total nitrogen utilization and amino acid synthesis. Moreover, AO090005001240 was expected to exhibit Xaa-Pro aminopeptidase. This enzyme can break down the Xaa-Pro peptide bond, which may contribute to the umami taste of soy sauce (13). In the future, more validation experiments, such as gene knockout or knock-in, can be conducted to demonstrate the significance of these genes.

In addition to identifying critical proteolytic enzyme genes, this work contributed to the phylogenetic distinction and carefully examined the molecular mechanisms between different industrial soy sauces. We discovered that A. oryzae LBM 30007 has greater proteolytic activity, which is consistent with the genetic information that strain LBM 30007 contains a large number of genes with signal peptides and increased protease transcription. In addition, we found that A. oryzae 3.042 has 10-fold and 64-folds more transcriptional expression of gene *brlA* (a conidiation regulator gene in A. oryzae) than that of LBM 30007 and LBM 30008, respectively, explaining why A. oryzae 3.042 can produce more spores.

This study provided high-quality genomic information on three industrial A. oryzae that are used in the production of Chinese and Japanese soy sauce. Using the high-quality genomic data, we investigated the evolutionary relationship between soy sauce industrial A. oryzae strains. The phenotypic characteristics of three industrial koji molds were determined. More importantly, we investigated the molecular mechanism behind the phenotypic differences. In the end, by integrating the phenotypic characteristics and transcriptional data, 58 candidate key proteolytic enzyme genes were identified. This study provided a thorough examination of the fermentative properties of Chinese and Japanese soy sauce koji molds, as well as a genetic and transcriptional examination behind these properties. This study identified candidate key proteolytic enzymes, thus helping with the generation of new strategies for higher soy sauce productivity and flavor quality. In addition, the results of this study may be advantageous for the development of key proteolytic enzymes, which might be useful for other protease-dependent industrial applications.

## MATERIALS AND METHODS

### Strains and culture.

Strains used in this work were obtained from the LBMAE (Laboratory of Brewing Microbiology and Applied Enzymology) collections at Jiangnan University. A. oryzae strains were inoculated on potato dextrose agar (PDA) medium and incubated at 30°C for 5 days. Before fermentation, the conidia of A. oryzae were collected as seeds and suspended in sterile water to make a conidia suspension of 10^8^ spores/mL for the study of fermentative characteristics or koji making. The mycelium was harvested for DNA extraction. Czapek-Dox agar (CDA) medium contains 0.3% NaNO_3_, 0.2% KCl, 0.1% KH_2_PO_4_, 0.05% MgSO_4_·7H_2_O, 0.002% FeSO_4_·7H2O, 2% glucose, and 1.5% agar, at pH 5.5. PDA medium was purchased from Sinopharm Chemical Reagent Co., Ltd, China.

### Study of fermentation characteristics.

To measure the conidia germination time of each industrial koji mold, 100 μL conidia suspension (10^7^ spores/mL) was spread on CDA medium and cultured at 30°C. Then, we observed whether the conidia germinated every hour (from 5 to 10 h) by using microscope equipment (BX51; Olympus). To measure the conidia quantity produced by each industrial koji mold, 100 μL conidia suspension (10^7^ spores/mL) was spread on PDA medium and cultured at 30°C for 5 days. Then conidia were gathered with sterile water from the 1st to the 5th day, and the number on each plate was determined by a hemocytometer. All of the above experiment was conducted in triplicate.

### Soy sauce koji fermentation.

To produce koji for soy sauce production, raw soybeans were soaked in warm water for 8 h and steamed at 120°C for 13 min. After the steamed soybeans cooled to ~40 to 50°C, wheat flour and steamed soybeans were mixed at a ratio of 1:4 (wt/wt) and inoculated with the prepared conidia suspension. Koji fermentation took about 40 to 48 h and could be divided into the following two stages: mycelial expansion and sporulation. At the hyphal expansion stage, koji was incubated at 30°C, with 95% humidity. During the sporulation stage, koji was fermented at 28°C with 80% humidity. Seed koji was produced by using 14 g of wheat bran, 6 g of wheat flour, and 14 mL of water in a 250-mL Erlenmeyer flask. Mixtures were autoclaved at 121°C for 20 min, cooled to 40°C, and inoculated with the freshly pure conidia suspension of A. oryzae and then cultivated at 30°C for 72 h.

### Physicochemical properties and enzymatic activities assay.

Total nitrogen and soluble nitrogen were determined by the Kjeldahl azotometer (Kjeltec 8000; FOSS) (39). Amino nitrogen was determined by using the formol titration method, according to the National Standard of the People's Republic of China GB5009.235-2016 (40). The content of glucose at different fermentation stages was measured by the SBA-40X biosensor (Biology Institute of Shandong Academy of Sciences, China). As previously reported, concentrations of amino acids in mature koji were determined by using a Hitachi amino acid analyzer (41).

For preparing a crude enzyme solution, 5 g of koji samples were mixed with 45 mL distilled water or the corresponding buffer and then stirred at 40°C for an hour. The protease activity was assayed by a modified method from the previous report, using pH 7.2 phosphate buffer (0.1 M Na_2_HPO_4_ plus 0.1 M NaH_2_PO_4_) for the neutral protease assay and pH 3.0 lactic acid buffer (0.1 M lactic acid plus 0.1 M sodium lactate) for the acid protease assay (42). One unit of protease was defined as the amount of enzyme that yields the color equivalent to 1 μg tyrosine in 1 mL of reaction solution per min at 40°C. The activity of amylase was determined according to the previous report with some modifications, using 0.8% starch solution dissolved in pH 5.9 phosphate buffer (0.05 M Na_2_HPO_4_ plus 0.05 M NaH_2_PO_4_) as substrate and measurement at 620 nm absorbance (43). One unit of amylase activity was defined as the amount of enzyme required to hydrolyze 1 mg soluble starch per min at 40°C. The activity of glucoamylase was determined according to the National Standard of the People's Republic of China QB/T1803-1999 as before (1). One unit of glucoamylase was referred to as the amount of enzyme that catalyzed the conversion of starch to 1 mg glucose per min at 40°C. Leucine aminopeptidase was determined as described in the previous report (44). One unit of leucine aminopeptidase was defined as the amount of enzyme that hydrolyzes leucine *p*-nitroaniline to produce 1 μg *p*-nitroaniline per min at 40°C. The method for detecting pectinase was according to the previous study (6). One unit of enzyme activity was defined as the amount of enzyme that hydrolyzes pectin to yield 1 μg galacturonic acid per min at 45°C.

All of the above experiments were conducted in triplicate. Significance analysis was determined by one-way analysis of variance (ANOVA) test and *post hoc*/multiple comparison test. *, *P* < 0.05; **, *P* < 0.01.

### DNA sequencing and genome assembly.

In a mortar, the mycelial mat was crushed into a fine powder using liquid nitrogen. Using the Ezup column fungi genomic DNA purification kit, genomic DNA was extracted (Sangon Biotech Shanghai Co., Ltd.) in accordance with the manufacturer's instructions and confirmed for integrity via agarose gel electrophoresis. Using a NanoDrop 2000 spectrophotometer (Thermo Scientific, USA), the purity of the DNA was assessed, and the concentration was analyzed by using a Quantus fluorometer (Promega, USA).

To promote the assembly level, a combination of next-generation sequencing (NGS) on the Illumina HiSeq platform and third-generation sequencing (TGS) utilizing Oxford Nanopore Technologies (ONT) was used. For Illumina X 10 sequencing, a paired-end library (150 bp) containing a 470-bp insert was constructed. After sequencing, the raw sequence data were processed by Trimmomatic v0.36 (45), SeqPrep (https://github.com/jstjohn/SeqPrep/) and Sickle v1.33 (https://github.com/najoshi/sickle/) to provide clean data for further analysis. The GridINO sequencers from ONT were used to carry out the TGS. The ligation sequencing kit SQK-LSK109 was used in accordance with the manufacturer's instructions to construct genomic libraries. According to the previous report (46), the raw genomic data from ONT were quality controlled and filtered.

To determine if the genome was contaminated and to assess the sequencing quality, the quality of the genome sequence obtained from the preliminary assembly was evaluated. Quality-filtered reads from the Illumina library were used to carry out GC depth distribution analysis by Bowtie2 v2.29 (https://sourceforge.net/projects/bowtie-bio/files/bowtie2/2.2.9/). In addition, a 17-mer frequency distribution was performed using Meryl to estimate the genome sizes according to the previous report (47). The *de novo* assembly of the clean reads was carried out using the SOAPdenovo v2.04 (48). Assembly evaluation was conducted by BUSCO v3.0 (49) and CEGMA v2.5 (http://korflab.ucdavis.edu/datasets/cegma/).

### Genome annotation and prediction.

RepeatMasker v4.0.7 (http://www.repeatmasker.org/) was used to find repeat sequences in three genomic scaffolds. All genomes were annotated by MAKER2 v2.31.9 (http://www.yandell-lab.org/software/maker.html), in which A. oryzae RIB40 was the reference to obtain coding sequences (CSD). Barrnap v0.8 (https://github.com/tseemann/barrnap/) and tRNAscan-SE v2.0 (50) were used to predict the rRNA and tRNA contained in the genome, respectively. The functional annotation of protein-coding genes was performed using NR (ftp://ftp.ncbi.nlm.nih.gov/blast/db/), eggNOG (http://eggnogdb.embl.de/#/app/home), Swiss-prot (http://uniprot.org), Pfam v31.0 (http://pfam.xfam.org/), GO (http://geneontology.org/), and KEGG (http://www.genome.jp/kegg/), with an e value of <1e−5.

Signal peptides were identified by SignalP v4.1 (http://www.cbs.dtu.dk/services/SignalP) with an e value of <1e−5.

### Gene family and phylogenetic tree construction.

Using OrthoFinder v2.4.1 (51), we categorized the orthogroups of proteins and found species-specific orthogroups. In addition to the three A. oryzae strains analyzed in this study, we searched the genomes of 19 other A. oryzae strains used in the soy sauce industry from the NCBI genome database. To construct a phylogenetic tree of soy sauce industrially utilized A. oryzae (22 strains) and seven additional sequenced fungi as the outgroups (see Table S1 in the supplemental material), proteins of single-copy orthogroups were selected and aligned using MAFFT (52). Then, Gblocks (https://home.cc.umanitoba.ca/~psgendb/doc/Castresana/Gblocks_documentation.html) was used to eliminate divergence and ambiguous alignment sites. Then, the trimmed alignment sequences were concatenated by SeqKit (https://bioinf.shenwei.me/seqkit/). After determining the optimal amino acid substitution model with IQ-TREE v1.6.12 (http://www.iqtree.org/), a maximum likelihood phylogenetic tree was built by IQ-TREE v1.6.12 with 1,000 bootstrap replicates. The phylogenetic tree was visualized using the iTOL web server (https://itol.embl.de/).

### Genome synteny analysis.

To identify the syntenic blocks in the whole genome, MCScan (https://github.com/tanghaibao/jcvi/wiki/MCscan-(Python-version)/) was used to analyze the genome synteny between A. oryzae LBM 30007, 3.042, LBM 30008, and RIB40 with the default parameters.

### Comparison of aflatoxin biosynthesis gene clusters.

A. flavus NRRL3357 is a typical strain of A. flavus that produces aflatoxin (24). The protein accession numbers for the members of the aflatoxin biosynthesis gene cluster in this strain are shown in Table S16 in the supplemental material. The predicted aflatoxin biosynthesis proteins in 22 A. oryzae genomes used in the soy sauce industry were aligned to those in A. flavus NRRL3357 by using the BLASTp function of the BLAST+ suite v2.9.0 with an e value of less than 10^−5^. For visualization of the gene cluster, EasyFig was used (53).

### Detection of the aflatoxin biosynthesis by UPLC-QQQ-MS.

In this study, aflatoxin was measured using the UPLC-QQQ-MS from Waters. The koji samples, which were cultivated by *Aspergillus* strains, were dried in a blast oven at 80°C. The materials were then crushed and screened using a 20-mesh filter. Five grams of the crushed material was added to 10 milliliters of ice water. Then, 15 mL of a solution containing 1.0% formic acid and acetonitrile were added to the solution (as an extracting agent). The above mixtures were shaken for 2 min rapidly on a vortex shaker. We added 1 g of NaCl and 4 g of anhydrous magnesium sulfate to the mixture, vigorously shook for 1 min, and then centrifuged at 8,000 rpm for 10 min. Supernatants of 6 mL were collected and were mixed with 900 mg of anhydrous magnesium sulfate. The above mixtures were centrifuged at 8,000 rpm for 10 min. Predetected samples were produced through a 0.22-μm organic filter membrane and generated the resulting filtrate.

For the UPLC condition, a C_18_ column (1.7 μm, 100 mm) was used for the detection of aflatoxin. For the mobile phase, 0.1% formic acid-water was used as mobile phase A, and 0.1% formic acid-methanol was used as mobile phase B. The flow rate was 0.3 mL min^−1^, and the injection volume was 10 μL. The gradient elution program was as follows: 0.00 min, 10% mobile phase B; 1.00 min, 10% mobile phase B; 3.00 min, 40% mobile phase B; 5.00 min, 60% mobile phase B; 9.00 min, 100% mobile phase B; 11.00 min, 100% mobile phase B; 12.00 min, 10% mobile phase B; MS condition, ion source was electrospray ionization source (ESI+) mode with a spray voltage of 3.5 kV(+); the ionization source temperature was 150°C, and the desolventizing temperature was 300°C. The parameters of mass spectrometry are listed in Table 3.

**TABLE 3:** Parameters of mass spectrometry

Aflatoxin	Precursor ion (*m*/*z*)	Daughter ion (*m*/*z*)	Cone voltage (V)	Collision energy (eV)
AFB1	313.16	285.09	22	40
AFB2	315.16	287.29	30	44
AFG1	329.07	199.85	42	36
AFG2	331.14	189.23	40	30

### SNP and InDel analyses.

BWA (Burrows-Wheeler alignment tool) (54) was used to map the high-quality filtered short reads of A. oryzae LBM 30007 and LBM 30008 to our A. oryzae 3.042 assembly. SAMtools v1.12 (55) was used to convert mapping results into BAM format and sort. Following the identification of duplicated readings, GATK4 v4.2.1.0 (https://anaconda.org/bioconda/gatk4) performed variant detection, SNP and InDel extraction, and filtering. SNPs and InDels annotation was performed by SnpEff v 5.0e (https://sourceforge.net/projects/snpeff/).

### RNA-seq analysis.

Spores and mycelia were collected in triplicate from each of the three fungi and crusted into powder in liquid nitrogen. The RNeasy Micro kit (Qiagen) was used to extract total RNA in accordance with the manufacturer's instructions. NanoDrop 2000 (Thermo Scientific, USA) was used to quantify the RNA’s purity and concentration, while gel electrophoresis and the 2100 bioanalyzer (Agilent Technologies, USA) were utilized to examine the RNA’s integrity. The qualified RNAs were sequenced using the Illumina NovaSeq 6000 system (Illumina Inc., San Diego, USA) by Majorbio Biopharm Technology Co., Ltd (Shanghai, China). We obtained gene expression profiles of several A. oryzae strains, including LBM 30007, 3.042, and LBM 30008, at different koji fermentation stages, namely, the mycelium expansion stage and the following sporulation stage.

For proteolytic enzyme gene expression analysis, the quality of reads was evaluated using fastp (https://github.com/OpenGene/fastp). SeqPrep (https://github.com/jstjohn/SeqPrep) and Sickle (https://github.com/najoshi/sickle) were utilized to remove adapters and low-quality reads. The clean RNA-seq reads were mapped onto the A. oryzae RIB40 genome (GenBank accession number GCF_000184455.2) (https://www.ncbi.nlm.nih.gov/genome/526) by Hisat2 v2.1.0 (http://ccb.jhu.edu/software/hisat2/index.shtml). Functional annotation to databases was the same as in “Genome annotation and prediction” above. The expression of annotated genes was quantified using RSEM v1.3.3 (http://deweylab.biostat.wisc.edu/rsem/), with transcripts per million reads (TPM) as an indicator. The differential expression analysis was done using DESeq2 v1.24.0 (http://bioconductor.org/packages/stats/bioc/DESeq2/), with the following parameters: *p*-adjust < 0.05, FC ≥ 2, and FC ≤ 0.5.

### Real-time PCR analysis.

Total RNA was extracted from three Aspergillus oryzae strains during the mycelium growth and sporulation stages. Then, cDNA was produced by removing genomic DNA and being reverse transcribed by using the HiScript III RT SuperMix (+gDNA wiper). cDNA was used as template for the fluorescent-based quantitative PCR by using the glyceraldehyde triphosphate dehydrogenase gene (AO090011000414/gapdh) as an internal reference. The real-time PCR system includes the following: AceQ Universal SYBR quantitative PCR (qPCR) master mix, 10 μL; template, 1 μL; upstream and downstream primers (10 μmol L^−1^), 0.4 μL; and double-distilled water (ddH_2_O), 8.2 μL. The reaction program was as follows: 95°C for 5 min; 95°C for 10 s, 60°C for 30 s, 40 cycles. The dissolution curve procedure was as follows: 95°C for 15 s, 60°C for 60 s, and 95°C for 15 s. Relative quantification used the 2^−ΔΔ^*^CT^* method. The primers used for real-time PCR are shown in Table 4.

**TABLE 4:** Primers used in real-time PCR

Gene	Primer	Sequence (5′→3′)
AO090003001036/ *alp1*	*alp1*-F	ATCTTCCTGTCGGTATTGAGC
	*alp1*-R	GACGTAGGCAACATCTTCGTTC
AO090011000036/ *NpI*	*NpI*-F	GCGAAGTATATCAATGCGACT
	*NpI*-R	AAATTGACATGAGCGACCC
AO090023000872	23000872-F	TTATTCCCTGCAATTCCACT
	23000872-R	TGATCGTCGAAACACAACCC
AO090011000942	11000942-F	GTCAATGCCACTCTTCGTT
	11000942-R	AGCGATCTCATATTCGTCT
AO090005001240	05001240-F	GACCGACGCTAACTATCGAG
	05001240-R	TAGCCGTTCATTATCTAGCC
AO090011000940	11000940-F	TCTACCCATATAACCGCAAC
	11000940-R	ATTTCCGTAGATCTTGCAT
AO090020000005	20000005-F	GACTCCTCTCCAAAACGGAA
	20000005-R	ACAAGGAAGCCTATTAGCAT
AO090011000414 /*gapdh*	*gapdh*-F	CGATTCTACCCACGGCAAC
	*gapdh*-R	TATTCCGCTCCAGCATCCTT

### Data availability.

Whole-genome sequences of 3 strains have been deposited in the NCBI GenBank database (https://www.ncbi.nlm.nih.gov/genbank/) under the accession number PRJNA800656. The transcriptome sequences are available in the NCBI Sequence Read Archive database (http://www.ncbi.nlm.nih.gov/sra/) under the accession number PRJNA801076.
