# Supplementary material for: Phenotypic, Genomic, and Transcriptomic Comparison of Industrial Aspergillus oryzae Used in Chinese and Japanese Soy Sauce: Analysis of Key Proteolytic Enzymes Produced by Koji Molds
Source: Microbiol Spectr. 2023 Feb 6;11(2):e00836-22. doi: 10.1128/spectrum.00836-22 (PMC10100866; doi:10.1128/spectrum.00836-22)
Supplement: Supplemental file 1 — Fig. S1 to S9. Download spectrum.00836-22-s0001.pdf, PDF file, 1.4 MB [file spectrum.00836-22-s0001.pdf]

Supplemental Figure

Phenotypic, genomic and transcriptomic comparison of industrial *Aspergillus oryzae* uesd in Chinese and Japanese soy sauce: analysis of key proteolytic enzymes produced by koji molds

Lijie Zhang#, Le Kang#, Yan Xu*

Laboratory of Brewing Microbiology and Applied Enzymology, Key Laboratory of Industrial Biotechnology of Ministry of Education, School of Biotechnology, Jiangnan University, 1800 Lihu Avenue, Wuxi, Jiangsu 214122, China

Running title: Comparative analysis of *A. oryzae* for key protease

#Both authors contributed to this manuscript equally

*Corresponding author. Tel.: +86-510-85918201; Fax: +86-510-85918201.
E-mail address: yxu@jiangnan.edu.cn (Yan Xu)

MATERIALS AND METHODS

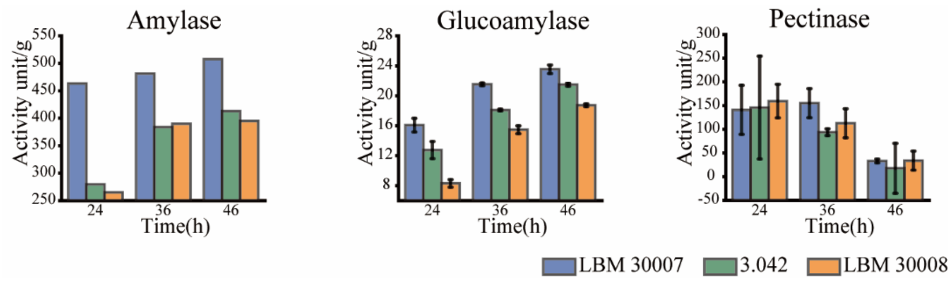

Fig S1. Carbohydrase activity of soy sauce koji during fermentation, using *A. oryzae* LBM 30007, 3.042, and LBM 30008 as the starter, respectively.

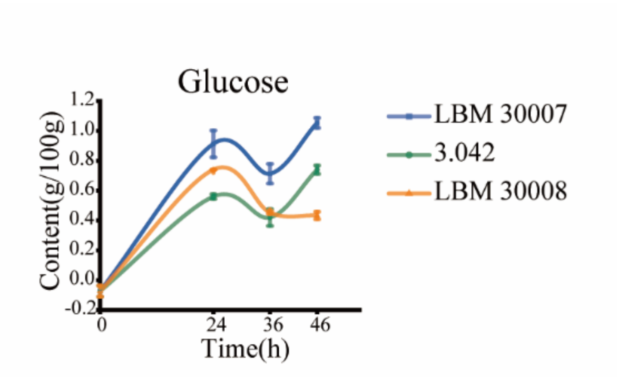

Fig S2. The content of glucose in soy sauce koji during the fermentation process, using *A. oryzae* LBM 30007, 3.042, and LBM 30008 as the starter, respectively.

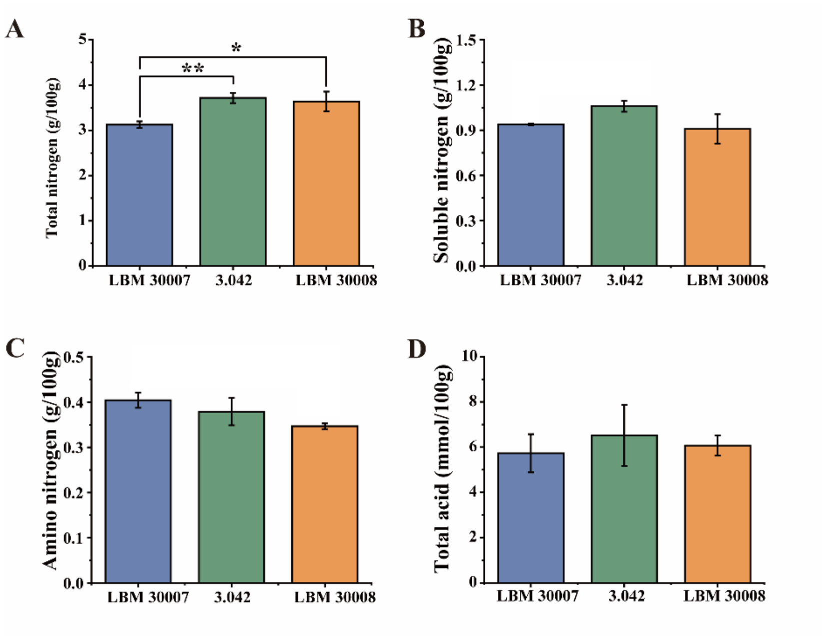

Fig S3. The significance analysis of fermentation parameters of various industrial strains.

Significance analysis was determined by one-way ANOVA test and post-hoc/multiple comparison test. *p < 0.05; **P< 0.01

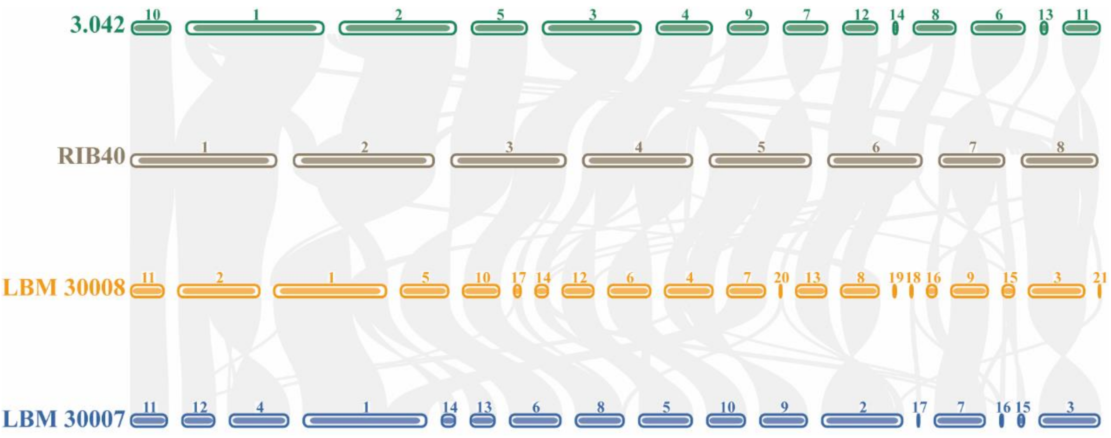

Fig S4. There are collinearity patterns between *A. oryzae* LBM 30007, 3.042, LBM 30008 and RIB40. The numbers indicate the scaffolding order. Each gray line represents a syntenic block. Scaffolds smaller than 60kb are not displayed.

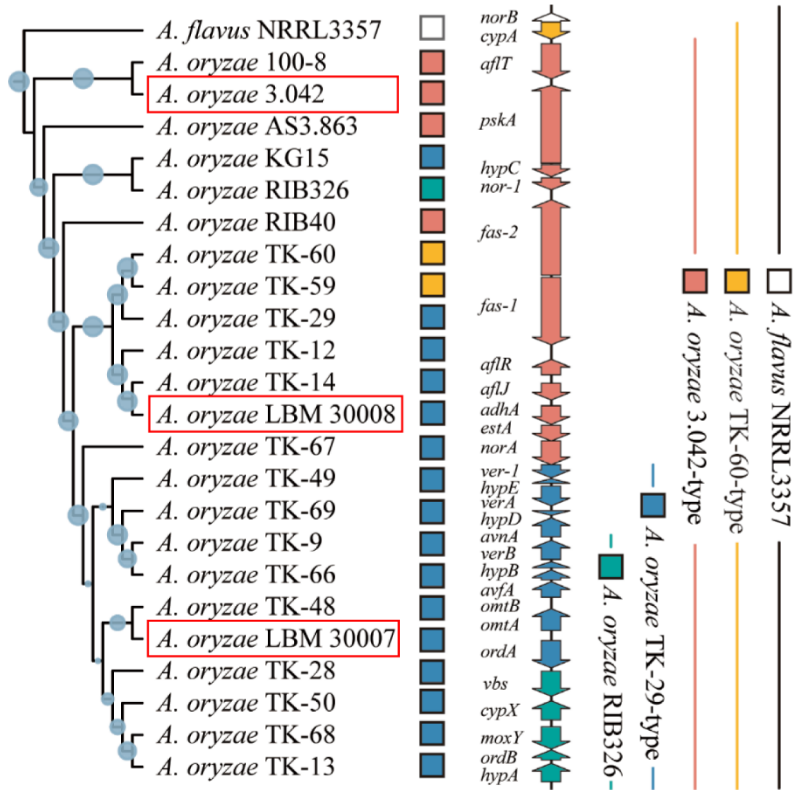

Fig S5. Comparison of aflatoxin biosynthesis gene clusters between *A. flavus* and industrial *A. oryzae*. The dendrogram represents the phylogenetic relationship between the 22 *A. oryzae* for soy

sauce brewing. *A. oryzae* RIB40 and *A. flavus* NRRL3357 are two type of strains. The size of the blue circles at the branches indicates the bootstrap support. Differently colored rectangles represent different types of aflatoxin biosynthesis gene clusters. Strains marked with green square mean that they are *A. oryzae* RIB326 type strains and has the aflatoxin biosynthesis gene clusters ranging from *vbs* to *hypA*. Strains marked with blue squares mean that they are *A. oryzae* TK-29-type strains and have the aflatoxin biosynthesis gene clusters from *ver-1* to *hypA*. Strains marked with red squares mean that they are *A. oryzae* 3.042-type strains and have the aflatoxin biosynthesis gene clusters from *aflT* to *hypA*. Strains marked with orange squares mean that they are *A. oryzae* TK-60-type strains and have the aflatoxin biosynthesis gene clusters from *cypA* to *hypA*. Genes that share >70% identity with those in *A. flavus* NRRL3357 are determined to be present and functional.

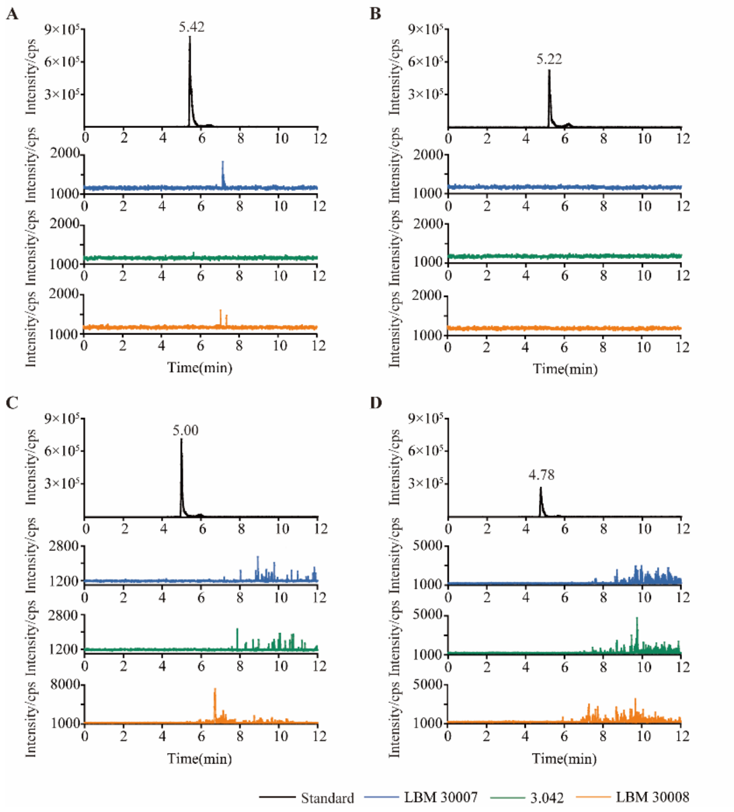

Fig S6. The detection of aflatoxin synthesis by *A. oryzae* LBM 30007, 3.042 and LBM 30008 at 24 h (A), 48 h (B), 72 h (C) and 96 h (D).

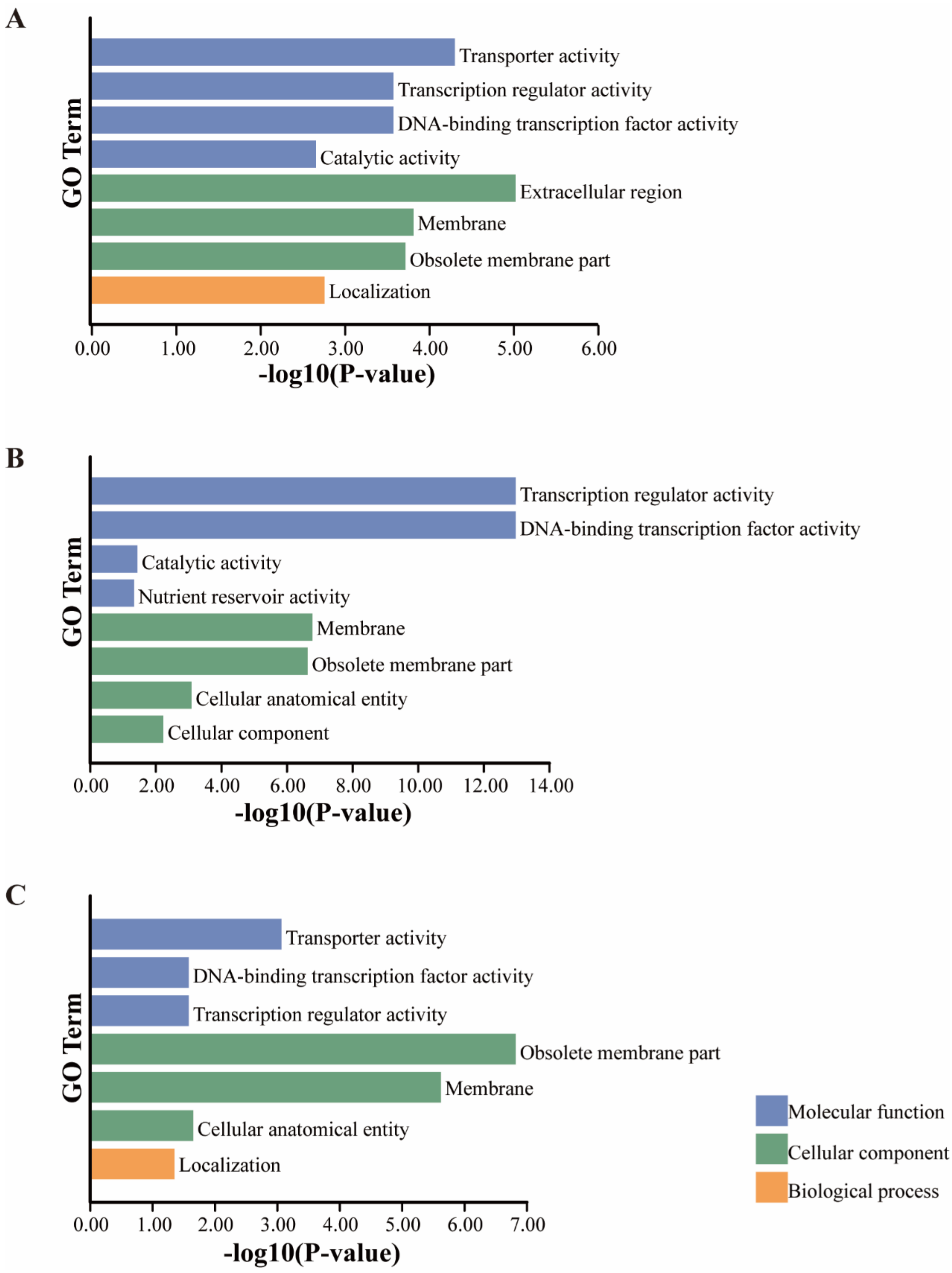

Fig S7. GO enrichment analysis of the SNP variant genes between Japanese *A. oryzae* LBM 30007 and LBM 30008 and Chinese *A. oryzae* 3.042: (A) Silent mutations; (B) Missense mutations; (C) Nonsense mutations.

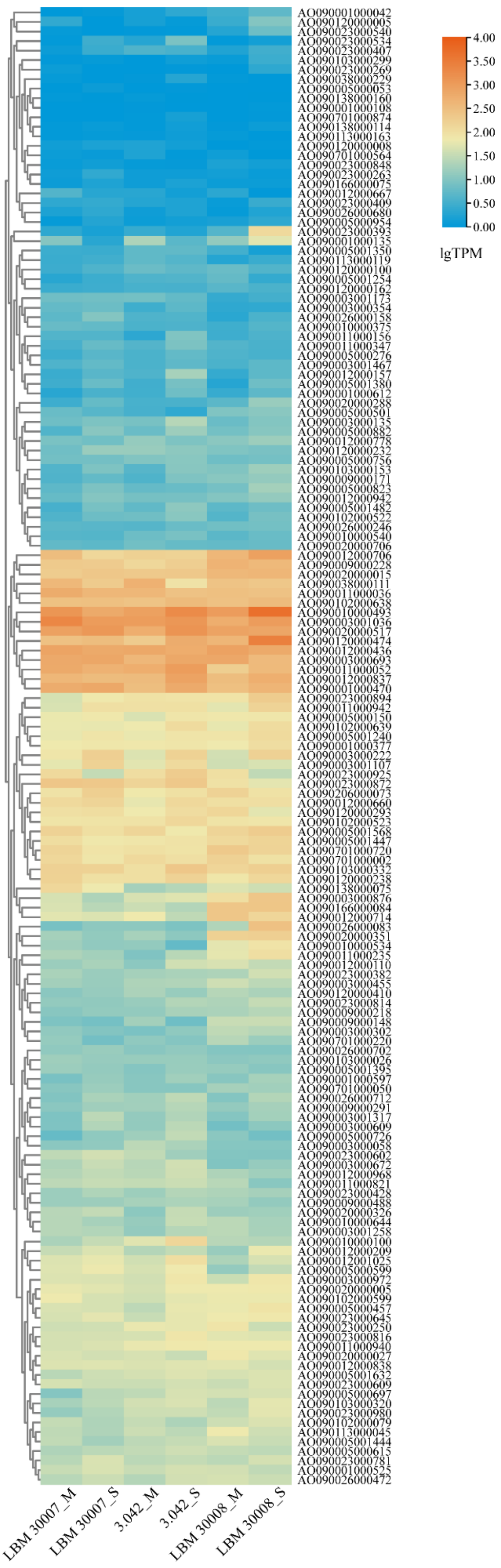

Fig S8. Heatmaps from hierarchical clustering of genes encoding proteolytic enzymes. M: Mycelium expansion stage, S: Sporulation stage.

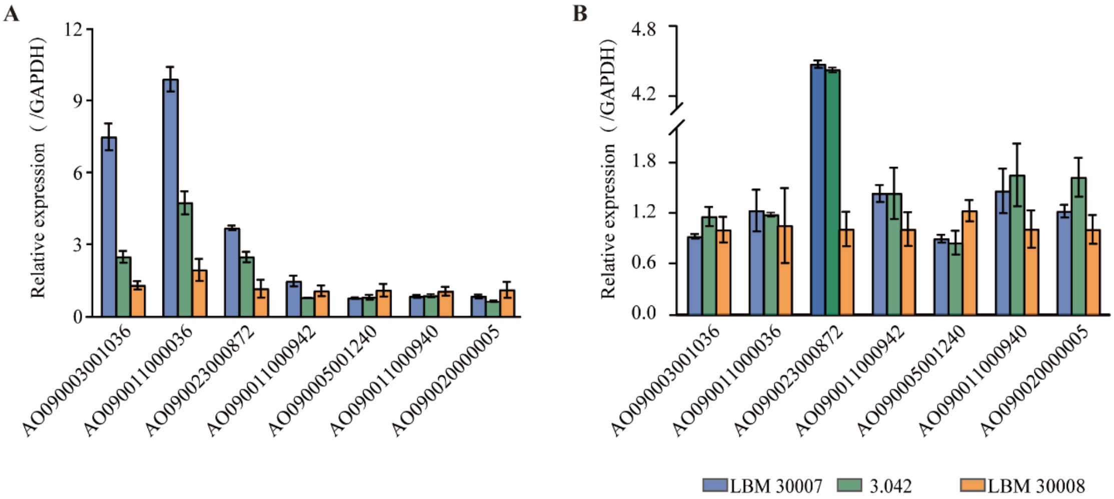

Fig S9. The transcriptional level of some genes was verified by qRT-PCR. A. Mycelium expansion stage, B: Sporulation stage.
